# Correction of the Electrical and Thermal Extrinsic Effects in Thermoelectric Measurements by the Harman Method

**DOI:** 10.1038/srep26507

**Published:** 2016-05-20

**Authors:** Min-Su Kang, Im-Jun Roh, Yun Goo Lee, Seung-Hyub Baek, Seong Keun Kim, Byeong-Kwon Ju, Dow-Bin Hyun, Jin-Sang Kim, Beomjin Kwon

**Affiliations:** 1 Center for Electronic Materials, Korea Institute of Science and Technology (KIST), Seoul, Republic of Korea 136-791; 2 Display and Nanosystem Laboratory, College of Engineering, Korea University, Seoul, Republic of Korea 136-713; 3 Department of Nanomaterials Science and Technology, Korea University of Science and Technology, Daejeon, Republic of Korea 305-333

## Abstract

Although the Harman method evaluates the thermoelectric figure-of-merit in a rapid and simple fashion, the accuracy of this method is affected by several electrical and thermal extrinsic factors that have not been thoroughly investigated. Here, we study the relevant extrinsic effects and a correction scheme for them. A finite element model simulates the electrical potential and temperature fields of a sample, and enables the detailed analysis of electrical and thermal transport. The model predicts that the measurement strongly depends on the materials, sample geometries, and contact resistance of the electrodes. To verify the model, we measure the thermoelectric properties of Bi_2_-Te_3_ based alloys with systematically varied sample geometries and either with a point or a surface current source. By comparing the model and experimental data, we understand how the measurement conditions determine the extrinsic effects, and, furthermore, able to extract the intrinsic thermoelectric properties. A correction scheme is proposed to eliminate the associated extrinsic effects for an accurate evaluation. This work will help the Harman method be more consistent and accurate and contribute to the development of thermoelectric materials.

Accurate evaluation of thermoelectric figure-of-merit, *ZT* = *α*^2^*T*/*ρk*, is of great importance to the development of thermoelectric materials, where *α* is the Seebeck coefficient, *ρ* is the electrical resistivity, *k* is the thermal conductivity, and *T* is the absolute temperature^1^,^2^,^3^,^4^,^5^,^6^. There are two common ways to determine *ZT*: first, independent evaluations of thermoelectric properties^4^,^6^,^7^,^8^,^9^; second, direct *ZT* measurements by the Harman method^1^,^2^,^5^,^10^,^11^,^12^,^13^. The first method is prevalent, since measuring each of the thermoelectric properties helps to understand the associated electrical and thermal transport phenomena, and the instruments for these measurements are more common. However, in this method, the total error combines the errors of the individual measurements, and it is, sometimes, difficult to measure along the same orientation of the sample. For example, in flash diffusivity method, the thermal diffusivity is measured in the out-of-plane direction^6^, whereas, in Van der Pauw method, the resistivity is evaluated in the in-plane direction^14^. On the other hand, the Harman method enables a rapid and simple *ZT* characterization, as this method calculates *ZT* based on the voltages (*V*) across the sample by an alternating current (AC) and a direct current (DC). Thus, the Harman method have become popular especially in industrial sectors where high-throughput screening is necessary.

However, to achieve high accuracy through the Harman method, many extrinsic factors should be considered such as thermal losses and electrical effects^2^,^5^,^10^,^12^,^15^. The Harman method obtains *ZT* from the relation *ZT* = (*V*_*DC*_/*V*_*AC*_ − 1)[*α*_*t*_*/*(*α*_*t*_ − *α*_*w*_)], where the subscripts “*t*” and “*w*” indicate the test material and lead wire, respectively. A crucial assumption underlying this relation is that Peltier effect at the junction between the lead wire and the material should dominate all the other thermal effects^10^. Thus, the instrument for the Harman method requires an adiabatic condition. Low gas pressure suppresses the convective heat flow and the use of thin and long lead wires reduces the heat conduction between the sample and the environment^2^,^5^,^10^,^15^. Small level of current minimizes the Joule heating of both the sample and the lead wires. In spite of these efforts, however, there still exist the Joule heating and the heat transfer via radiation and conduction^2^,^5^,^15^. Especially, the heat transfer from or to the sample reduces the temperature difference across the sample (Δ*T*), leading to the reduction of *V*_*DC*_, and the underestimation of *ZT*. Furthermore, *ZT* measured by Harman method largely depends on the sample size.

There have been efforts to correct the size effect. Theoretical studies have revealed the relation between the intrinsic *ZT* (*ZT*_*i*_) and measured *ZT* (*ZT*_*m*_) considering the thermal effects^2^,^5^,^12^,^13^,^15^,^16^. The theoretical relation suggests that the thermal effect becomes smaller when the sample shape factor, defined by the sample length (*L*) divided by cross-section area (*A*), is reduced. Thus, it seems to be possible to obtain *ZT*_*i*_ by measuring *ZT*_*m*_ as a function of *L*/*A*, and extrapolating *ZT*_*m*_ to *L*/*A* = 0 5. However, not only thermal effects but also electrical effects become important when *L*/*A* becomes smaller. For a sample with small *L*/*A*, the electrical resistance is small, thus the contact resistance associated with the voltage probes may add non-negligible contribution^2^. Moreover, for a sample with *A* much larger than the cross-section area of lead wires, the current density may be non-uniform over a finite length due to the current crowding.

In this study, we investigate the electrical and thermal extrinsic effects associated with the Harman method, and seek to obtain the intrinsic *ZT* from measured *ZT*. First, we develop a finite element model that helps to understand the contributions of each of the extrinsic effects. Then, by measuring three types of thermoelectric materials with various shape factors, we verify the developed model, and show how the accuracy of the Harman method may be improved.

## Overview of Harman Measurement

In our Harman measurement system, a sample is suspended by two pairs of lead wires in a vacuum chamber (10^−4^ Torr)^5^. The wires are ~20 mm long with a diameter of either 25 μm (Au wire) or 50 μm (Pt wire). A pair of lead wires are attached at the sample end surfaces to pass an electric current (25 mA). Figures 1(a) and 2(a) show two attachment configurations: (1) spot-welding of wires directly on a sample; (2) soldering of wires on Cu foils (thickness of ~500 μm) which are attached to the sample with electrically conducting paste. We employed an epoxy with dispersed silver particles which is assumed to have several orders of magnitude lower effective electrical resistivity as compared to the Bi-Te based alloys^12^. Nevertheless, it is desirable to distribute the conducting paste uniformly across the sample surface to avoid non-uniform electrical current distribution. Another pair of lead wires, which is spot-welded within the sample, measures the electrical potential variation. The positions of the voltage probes affect the measurement precision, which will be discussed below. Measurement of *V*_*AC*_ provides measured electrical resistivity (*ρ*_*m*_), and an additional measurement of *V*_*DC*_ gives *ZT*_*m*_. To estimate the uncertainties in both *ρ*_*m*_ and *ZT*_*m*_, we measured a single sample 10 times using the electrode configuration with Cu foils. For each measurement, the Cu foils and voltage probes were reattached.

Table 1 lists three types of test materials used for this study. The methods to obtain physical properties of the test materials are described in the Section 3. The test samples are Bi_2_-Te_3_ based sintered materials that were prepared in our group either via hot-extrusion technique (type 1 and 2)^17^, or spark plasma sintering (type 3)^18^. To vary the shape factor, we modified either *L* or *A*. For type 1 and 2, *L* was 35.4 mm for the first measurement. Then, the samples were cut to shorter lengths, keeping the same area, and further measurements were carried out. To see the influence of the thermal conduction, Joule heating, and Peltier effect along the lead wires, the wire material was chosen as either Au (*k*_*Au*_ = 314 W/mK, *ρ*_*Au*_ = 2.26 μΩcm, *α*_*Au*_ = 1.94 μV/K)^19^,^20^ or Pt (*k*_*Pt*_ = 71.6 W/mK, *ρ*_*Pt*_ = 10.4 μΩcm, *α*_*Pt*_ = −5.15 μV/K)^19^,^20^. Thus, as compared to Pt, if all the wires have the same dimension, Au roughly has ~4.4X larger thermal loss, ~4.6X smaller Joule heating, and smaller Peltier effect against Cu foil (*α*_*Cu*_ = 1.83 μV/K). For type 3, *A* was 43.6 mm^2^ for an initial measurement. Then, the sample was cut to smaller area, maintaining the same length, and additional measurements were made.

## Finite Element Model

We developed a three-dimensional thermoelectric finite element model (FEM) using a commercial software package (COMSOL Multiphysics) for the Harman measurement system. This FEM is useful to capture the non-uniform electrical and thermal transport between the lead wires and the sample, which arise from a large mismatch of the cross-section areas. Considering that the typical cross-section area of a sample is ≥1 mm^2^, the wire has ~1000X smaller cross-section area, which results in the spreading or crowding of the current or heat flow over a finite sample length.

For the simulation of the DC measurement, the FEM solves for both the thermoelectric, electrical, and thermal equations. The governing equation for heat flux, ***q***, in solid-state materials is ***q*** = −*k*∇*T* + *αT**J***, where ***J*** is the current density. The governing equation for current density is ***J*** = −(Δ*V* + *α*∇*T*)/*ρ*. The model includes a radiative heat flux (*q*_*rad*_) based on a relation *q*_*rad*_ = *εσ*(*T*^4^ − *T*_0_^4^), where *ε* is the effective emissivity, *σ* is the Stefan-Boltzmann constant, and *T*_*0*_ is an ambient temperature which is assumed as 298 K. As a thermal boundary condition, all the end surfaces of the lead wires maintains *T*_*0*_. As an electrical boundary condition, the end surfaces of the voltage probes are insulated. One of the current source wires supplies 25 mA, while the counter-side current source wire serves as a ground electrode. For the simulation of the AC measurement, the FEM solves for only the electrical governing equation which is ***J*** = −Δ*V*/*ρ*.

The model simulates the Harman measurement by calculating the thermal and electrical potential distributions over the sample and lead wires. The model estimates the voltage between the voltage probes, and gives the simulated *V*_*AC*_ (denoted as *V*_*AC,s*_) and *V*_*DC*_ (denoted as *V*_*DC,s*_). Then, the measured resistivity can be simulated by the following relation.


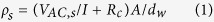


where *I* is the electric current, *R*_*c*_ is the contact resistance associated with the voltage probes, and *d*_*w*_ is the distance between the voltage probes. Similarly, the measured *Z* can be simulated by *Z*_*s*_ = (*V*_*DC,s*_/*V*_*AC,s*_ − 1) [*α*_*t*_*/*(*α*_*t*_ − *α*_*w*_)]/*T*_*0*_. For the calculations, the model requires the input values of *α*,*ρ*, and *k* of all the materials. Literature provides the properties of the lead-wire materials^19^,^20^. Table 1 shows the input Seebeck coefficients of the test materials measured through a static DC method. To find appropriate input values of *ρ* for the test materials, we fit *ρ*_*s*_ to the measured resistivity (*ρ*_*m*_) for all the sample shapes and current source types. Particular combination of input *ρ* and *R*_*c*_ provides the best fit. Likewise, to determine proper input values of *k* for the test materials, we fit *Z*_*s*_ to the measured *Z* (*Z*_*m*_). Since *V*_*DC,s*_ depends on the choice of *k*, a certain input *k* gives the best fit. Table 1 shows all the fitted properties, and data for the fitting procedures will be shown below.

## Results and Discussion

The developed FEM calculated the electric potential and temperature fields of the test samples under the Harman measurement condition. The calculation results are useful to understand the extrinsic effects and their contributions on the measurement accuracy. By fitting the measured and calculated data, the intrinsic values of the thermoelectric properties and contact resistance are determined, and the errors due to the extrinsic effects are uncovered.

Figures 1 and 2 show the simulated electric potential and temperature fields of type 3 sample under DC measurement. The data are captured in the symmetry plane to qualitatively assess the electrical and thermal transport. When electric current passes from a thin lead wire directly to a large sample, the lead wire acts as a point current source. The electric potential and the temperature change radially over a finite length, indicating that the current and heat flow spread out to the large region. Hence, there exist a regions where the electrical and temperature fields are not one-dimensional. This effect is relatively enormous for the sample with a small shape factor. On the contrary, when electric current enters the sample through a sufficiently thick layer of highly conductive material, the conductive layer serves as a surface current source. The calculation shows that a 500 μm-thick Cu layer well distribute the current and heat flow such that the electric potential and temperature fields are one-dimensional throughout the sample. Therefore, one-dimensional Ohm’s law and Fourier heat conduction law are well suited with the Harman measurement with a surface current source.

Not only the current source type but also the voltage probe position affects the Harman measurement. Figure 3 shows the resistivity as a function of the distance between the voltage probes (*d*_*w*_), measured with the surface current source. The data for the two types of samples show that measured resistivity become more consistent and smaller with larger *d*_*w*_. When *d*_*w*_ is too small (≤10 mm), the contribution of the error in *d*_*w*_ measurement becomes prominent. Furthermore, when the measured voltage is small (≤mV), the electrical extrinsic effects including the contact resistance may add more errors in the voltage measurement. Thus, we attached the voltage probes within few mm apart from the sample end surfaces for the following measurement in order to make *d*_*w*_, equivalently the sample resistance, as large as possible.

Figure 4 shows the resistivity of the test samples as a function of the sample shape factor. When *L*/*A* approaches to 0, the resistivity decreases rapidly with a point current source, while increases with a surface current source. With an appropriate *ρ* and *R*_*c*_ input in the FEM, the measured resistivity is well simulated. *R*_*c*_ = 0.3 mΩ and *ρ* denoted as intrinsic value in the figure provide the best fit. When *L*/*A* is below 1, the sample resistance is usually few mΩ, thus *R*_*c*,_which is nearly consistent regardless of *L*/*A*, becomes non-negligible in our system. Accordingly, the resistivity measured with a surface source is not constant, and becomes larger with a smaller *L*/*A*. On the other hand, with a point source, the measurement is significantly affected by the non-uniform current density when *L*/*A* is below 1. In this case, the measured resistivity is, sometimes, more than 50% less than the estimated intrinsic resistivity.

There are several ways to avoid the effect of the contact resistance. Based on the data in Fig. 4, measuring a sample with large enough resistance (≥100X of *R*_*c*_) almost eliminates the influence of *R*_*c*_. Another method is to estimate *R*_*c*_ by fitting the measured resistivity with Eq. 1 for various samples. The estimated *R*_*c*_ can be subtracted from the measured resistance. Moreover, when the resistance is measured with two samples of different *L*/*A*, *R*_*c*_ can be removed by subtracting those resistance each other (known as differential method). For example, when there are two samples with (*L*/*A*)_1_ and (*L*/*A*)_2_, the difference of the resistance (Δ*R*) is expressed as


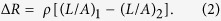


Equation 2 evaluates *ρ* without the knowledge of *R*_*c*_. Note that all these approaches work with the measurement with a surface current source.

Figure 5 shows the resistivity of the test samples subtracted by an effective contact resistivity (*ρ*_*c*_). *ρ*_*c*_ is simply defined as *R*_*c*_*A*/*d*_*w*_. For the data by a surface current source, *ρ* − *ρ*_*c*_ obtained either by directly subtracting *ρ*_*c*_ or by differential method show consistent values to each other and to the estimated intrinsic resistivity. However, when *L*/*A* is extremely small (<0.5), *ρ* − *ρ*_*c*_ still fluctuates, suggesting that sufficiently large *L*/*A* ensures the precise measurement. For the data by a point current source, *ρ* − *ρ*_*c*_ still strongly depends on *L*/*A* due to the effect of non-uniform current density.

Figure 6 shows *Z* of the test samples as a function of the sample shape factor. When *L*/*A* approaches to 0, *Z* increases almost linearly and suddenly drops when *L*/*A* becomes smaller than 1. With a proper *k* input in the FEM, the simulated figure-of-merit (*Z*_*s*_) fits well the measured data. *k* value for the best fit enables the estimation of the intrinsic *Z*. The linear dependence of *Z* on *L*/*A* is due to the thermal effects such as radiative heat transfer, conductive heat flow through wires, and Joule heating occurring within both the wires and sample^2^,^5^,^12^. If the thermal effects are only dominant extrinsic factors for the Harman measurement, *Z* should keep increasing until *L*/*A* reaches 0. However, for the sample with a small *L*/*A*, the influence of the contact resistance becomes apparent, and causes the sudden decrease of *Z*. For instance, assuming *R*_*c*_ = 0, *Z* is obtainable from *Z* = (*V*_*DC*_/*V*_*AC*_ − 1) [*α*_*t*_*/*(*α*_*t*_ − *α*_*w*_)]/*T*. However, if *R*_*c*_ is non-zero, it adds a positive offset to both sides of the fraction as *Z* = [(*V*_*DC*_ + *IR*_*c*_)/(*V*_*AC*_ + *IR*_*c*_) − 1] [*α*_*t*_*/*(*α*_*t*_ − *α*_*w*_)]/*T*, and reduces *Z*. Thus, to avoid the influence of *R*_*c*_, the sample should possess sufficiently large resistance, which is possible with a large *L*/*A*. For Bi-Te based alloys, *L*/*A* > ~1.5 have ensured enough resistances over *R*_*c*_. If a test material possesses small resistivity, proper range of *L*/*A* should be found based on the dependence of *Z* on *L*/*A*. It was also observed that for a single sample with *L*/*A* = 3.4, the uncertainty of measurement results was ±1.4% for *ρ*_*m*_ and ±2.2% for *ZT*_*m*_ which ensured an accuracy up to the second decimal point. These uncertainties would result from the uncertainties in the probe distance measurements, electrode qualities, or the inconsistent thermal loss due to different wire length or the fluctuation of surrounding temperature.

Differences between the point and surface current sources originate also from the thermal effects. With the surface source, heat flow across the sample end surface is also efficient, thus the conductive thermal loss through the lead wires tends to be large. Thus, for type 1 and 2, *V*_*DC*_ was smaller for the surface source than for the point source. However, when the cross section area of the sample is much larger (≥5000X) than that of the lead wire, *V*_*DC*_ was larger for the surface source than for the point source as shown in type 3. With the point source, heat due to the Peltier effect at the lead wire-sample junction does not efficiently flow across the sample end surface, resulting non-uniform temperature distribution. Thus, Δ*T* between the voltage probes may further reduce when the sample cross-section area is too large. In spite of the non-uniformity problem with the surface source, *Z* and its dependence on *L*/*A* were not greatly different between the measurements with the point and surface sources. This fact indicates that the effects of non-uniform fields in measured *V*_*DC*_ and *V*_*AC*_ roughly cancel each other. However, it is evident that the surface source must be more reliable, since it ensures the one-dimensional electrical potential and temperature fields, which are compatible with the Harman relation and its correction method.

By correcting both the electrical and thermal extrinsic effects, intrinsic *Z* (*Z*_*i*_) is obtainable from the Harman measurements. First, the electrical effects are corrected by measuring with surface current source, and subtracting *R*_*c*_. *Z* that is corrected for *R*_*c*_ (denoted as *Z*_*c*_) is acquired by


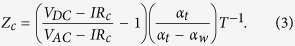


Note that, for the materials with *α*_*t*_ ~ 200 μV/K, *α*_*t*_*/*(*α*_*t*_ − *α*_*w*_) corrects for 1–2% of initially measured *Z*. If there exists an error when evaluating *α*_*t*,_denoted as *ε*, the term for the Seebeck coefficients can be expressed as *α*_*t*_(1+ *ε*)*/*[(*α*_*t*_(1+ *ε*) − *α*_*w*_)] = *α*_*t*_*/*[(*α*_*t*_ − *α*_*w*_/(1+ *ε*)]. Thus, if *α*_*w*_ is ~1 μV/K, fortunately the influence of *ε* would not be great. Then, the thermal effects are corrected by measuring multiple samples with various shape factors, and extrapolating the data to *L*/*A* = 0. Based on the previous study that only corrects for the thermal effects^5^, the relation between the *Z*_*c*_ and *Z*_*i*_ is expressed as


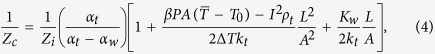


where *β* is the radiative heat transfer coefficient, *P* is sample perimeter, 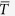 is an average temperature across the sample, and *K*_*w*_ is the thermal conductance of lead wires. According to Eq. 4, 1/*Z*_*c*_ is a second order polynomial of *L*/*A*, and 1/*Z*_*i*_ is obtainable at the y-axis intercept of the polynomial. To simplify Eq. 4, we assume Δ*T* is a linear function of *L*/*A* such as Δ*T* = *cL*/*A*, where *c* is a constant. This assumption is not too ideal based on the experimental data where Δ*T* is estimated from Δ*T* = (*V*_*DC*_ − *V*_*AC*_)/(*α*_*t*_ − *α*_*w*_). Furthermore, near room temperature or when the sample is in thermal equilibrium with surrounding radiation shield, we may assume 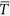 ~ *T*_*0*_. Then, Eq. 4 becomes


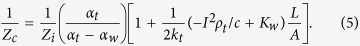


In the simplified relation, 1/*Z*_*c*_ is a linear function of *L*/*A*.

Figure 7 shows 1/*Z*_*c*_ of the test samples as a function of the shape factor. Within a particular range of *L*/*A* (~1.5 ≤ *L*/*A* ≤ ~4), 1/*Z*_*c*_ is a linear function of *L*/*A*, indicating that the radiative loss is not significant near room temperature. Even at high temperature, the linearity between 1/*Z*_*c*_ and *L*/*A* would not break if the sample and the surrounding radiation shield possess similar temperatures. Thus, when linearly extrapolating the data measured with surface source, y-axis intercepts are almost identical to *α*_*t*_*/Z*_*i*_(*α*_*t*_ − *α*_*w*_). The slope of the linear trend depends on Joule heating and conductive heat flow through lead wires as dictated by Eq. 5. To estimate the influence of the heat conduction, Eq. 5 was fitted with the measured data by employing *K*_*w*_ as a fitting parameter. For the best fit, *K*_*w*_ was chosen between 100 μW/K and 350 μW/K. Note that the slopes are different for different materials, measuring temperature and wiring conditions, as *ρ*, *k*, and *K*_*w*_ would not the same. Interestingly, extrapolating the data acquired with the point source also provides similar y-axis intercepts for type 1 and 2, although it exhibited a large discrepancy for type 3.

To simply see the influence of the current source on the Harman measurement, the FEM calculated several possible conditions. Figure 8 shows the simulated figure-of-merit (*Z*_*s*_) of type 3 with several configurations. When the contact resistance (*R*_*c*_) is not corrected, both the point and surface sources possess large error when *L*/*A* is small. However, without *R*_*c*_, *Z*_*s*_ becomes larger, is proportional to 1/(*L*/*A*)^~1^, and the extrapolation of *Z*_*s*_ to *L*/*A* = 0 gives the intrinsic *Z* (=2.40). When the lead wire material is Cu, the Peltier effect between the lead wire-Cu foil interface can be excluded and the Joule heating within the wire can be minimized. However, the Cu wire facilitates the conductive heat flow such that *Z*_*s*_ becomes smaller.

## Conclusions

We studied the electrical and thermal extrinsic effects on the Harman method. If the sample has a large shape factor (*L*/*A*), the thermal effects such as radiative heat transfer, conductive heat flow through lead wires, and Joule heating primarily affect the figure-of-merit (*Z*) measurement, and induce a linear dependence of 1/*Z* on *L*/*A*. However, if the sample shape factor is small (equivalently small electrical resistance), the electrical effects such as contact resistance and non-uniform current flow significantly deform the trend of *Z*. Therefore, it is important to correct all those extrinsic effects in the Harman method to accurately evaluate the intrinsic *Z*. The use of surface current source could ensure one-dimensional electric potential and temperature fields. In addition, the contact resistance could be determined through resistivity measurements or subtracted by a differential method. After correcting the electrical effects, extrapolation of measured 1/*Z* to *L*/*A* = 0 could correct the thermal effects, and provide the intrinsic *Z*. For our system, a particular range of *L*/*A* (~1.5 ≤ *L*/*A* ≤ ~4) provided accurate results, as the sample resistance was too small with small *L*/*A* and the sample was probably too heavy for the lead wires with large *L*/*A*. This work will help to enhance the accuracy of thermoelectric evaluation by the Harman method, and to develop better thermoelectric materials.

## Additional Information

**How to cite this article**: Kang, M.-S. *et al*. Correction of the Electrical and Thermal Extrinsic Effects in Thermoelectric Measurements by the Harman Method. *Sci. Rep*. **6**, 26507; doi: 10.1038/srep26507 (2016).

## Figures and Tables

**Figure 1:**
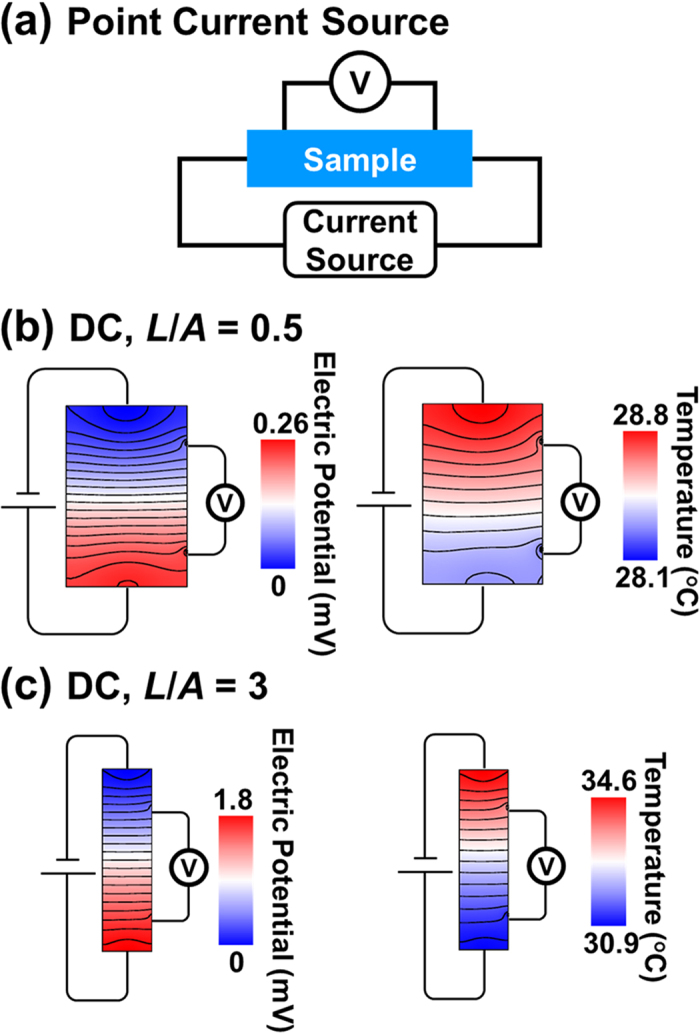
(**a**) A schematic for Harman method using a point current source. Calculated electric potential and temperature distributions in a sample when (**b**) *L*/*A* = 0.5 and (**c**) *L*/*A* = 3. For this calculation, the sample is subject to a DC current of 25 mA. Lines correspond to equipotential lines in the electric potential distributions and isothermal lines in the temperature distributions.

**Figure 2:**
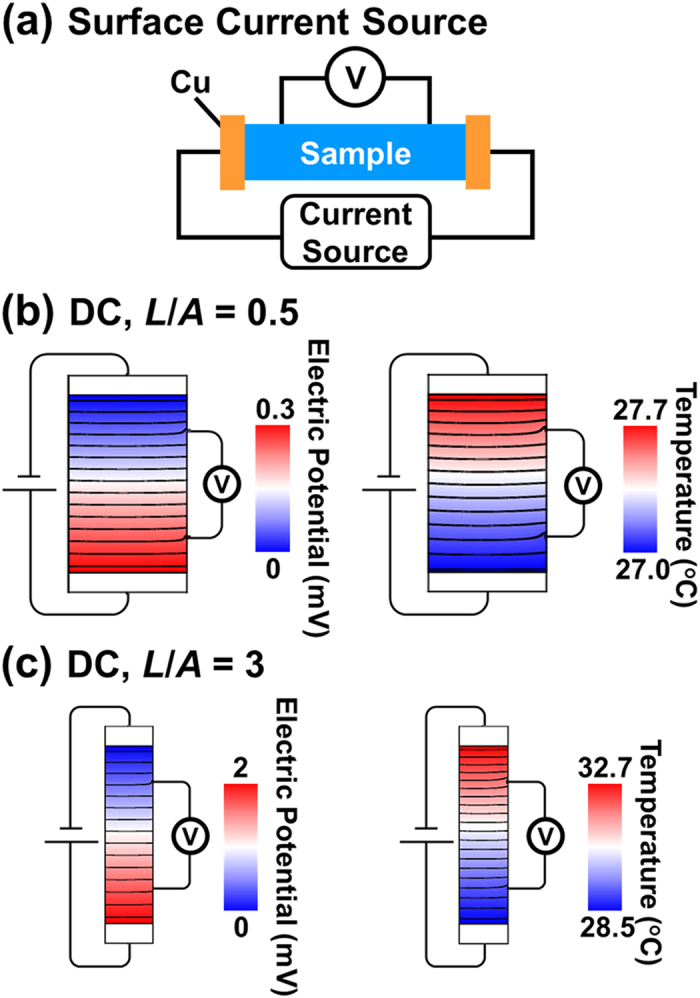
(**a**) A schematic for Harman method using a surface current source. Calculated electric potential and temperature distributions in a sample when (**b**) *L*/*A* = 0.5 and (**c**) *L*/*A* = 3. For this calculation, the sample is subject to a DC current of 25 mA. Lines correspond to equipotential lines in the electric potential distributions and isothermal lines in the temperature distributions.

**Figure 3:**
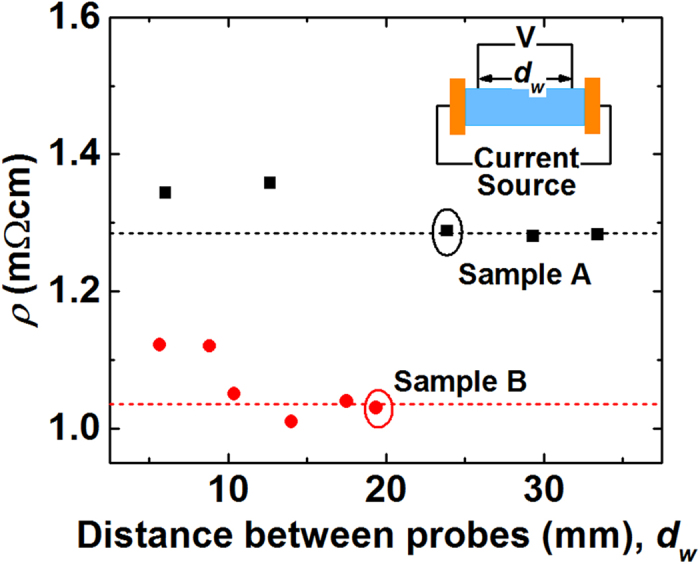
Resistivity measured with various distances between the voltage probes. The distance between the voltage probes has been chosen as ≥~5 mm.

**Figure 4:**
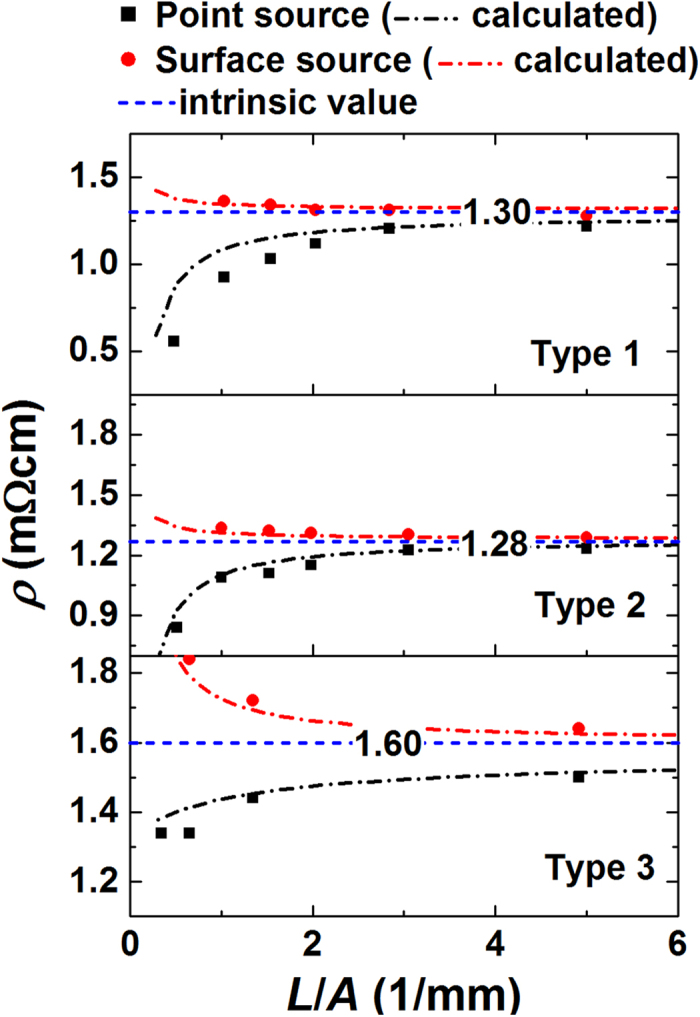
Resistivity of three types of Bi_2_-Te_3_ based materials. Measured resistivity (symbols) change as a function of the sample shape factor. Calculated resistivity (dash-dot lines) were fit to the measured resistivity by adjusting contact resistance and intrinsic resistivity (dash lines).

**Figure 5:**
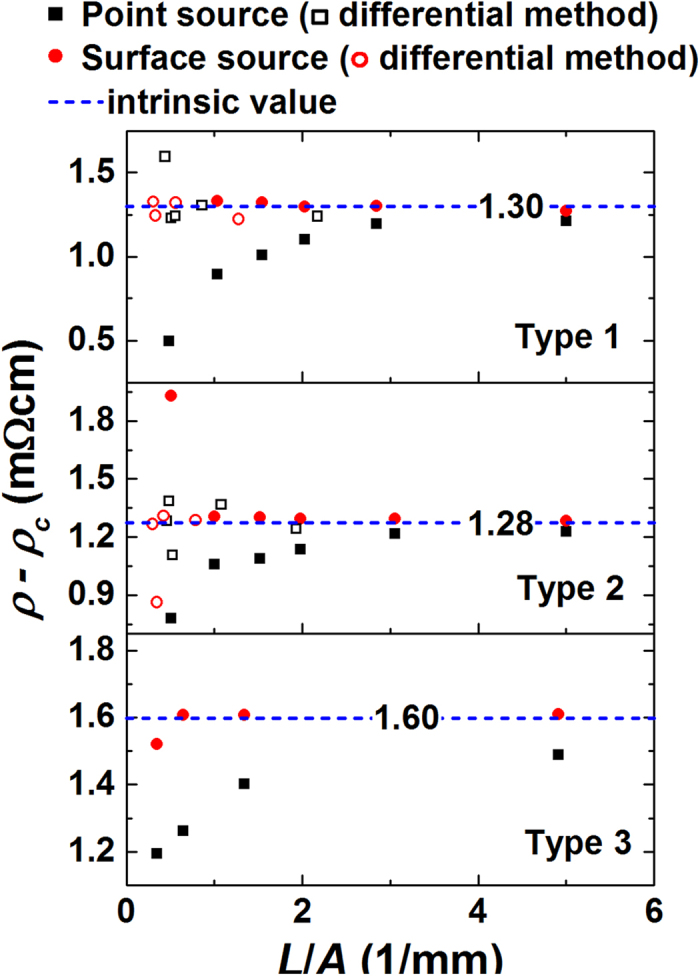
Resistivity of three types of Bi_2_-Te_3_ based materials subtracted by an effective contact resistivity, *ρ*_*c*_. Effective contact resistivity can be eliminated either directly subtracting *ρ*_*c*_ (filled symbols) or subtracting another resistivity (open symbols) from a particular resistivity. Intrinsic resistivity serve as reference (dash lines).

**Figure 6:**
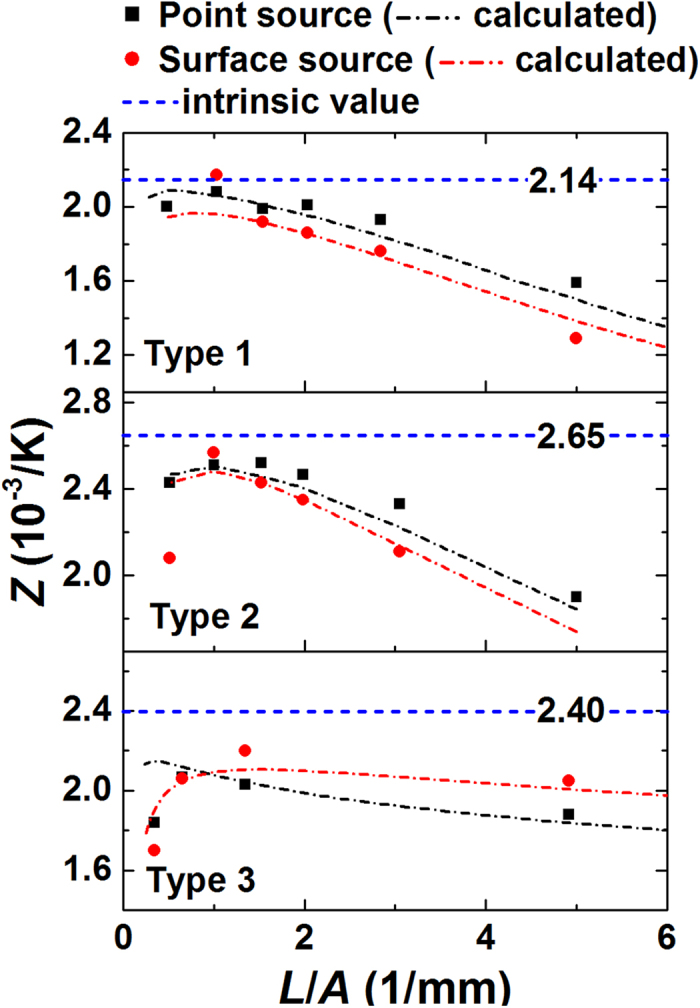
*Z* of three types of Bi_2_-Te_3_ based materials. Calculated *Z* (dash-dot lines) were fit to the measured *Z* (symbols) by adjusting the intrinsic thermal conductivity. The fitted thermal conductivity enables the calculation of intrinsic *Z* (dash lines).

**Figure 7:**
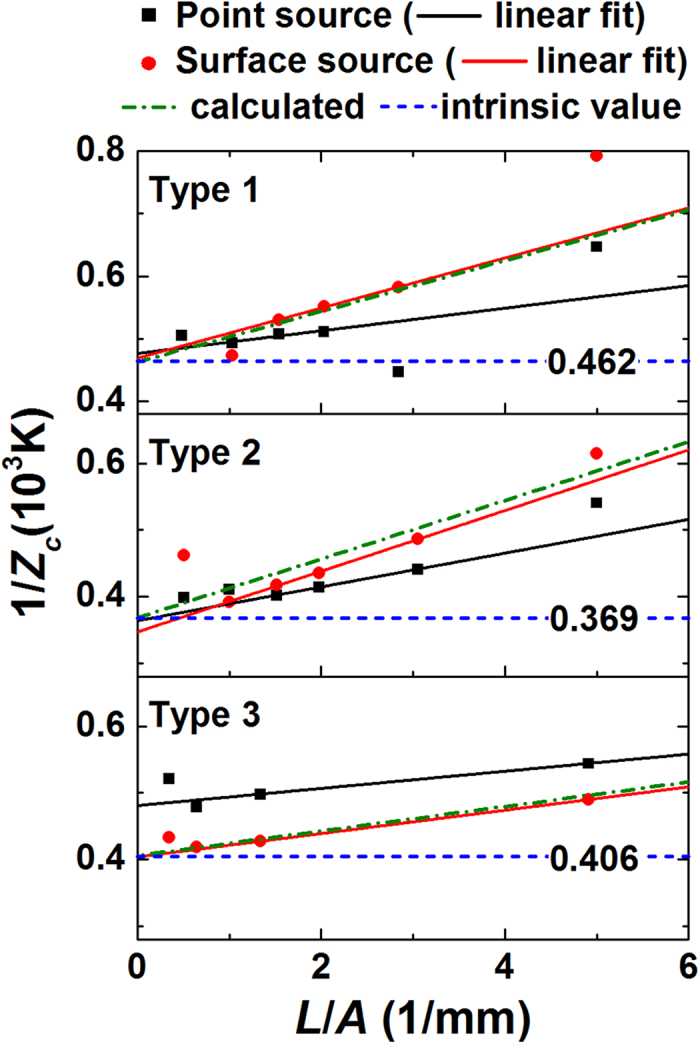
1/*Z*_*c*_ of three types of Bi_2_-Te_3_ based materials which were corrected for the contact resistance. Calculated *Z*_*c*_ (dash-dot lines) were fit to the measured *Z*_*c*_ (symbols) by adjusting the thermal conductance of lead wires. The y-axis intercepts of the calculated *Z*_*c*_ correspond to the intrinsic 1/*Z* multiplied by [*α*_*t*_/(*α*_*t*_ − *α*_*w*_)] (dash lines).

**Figure 8:**
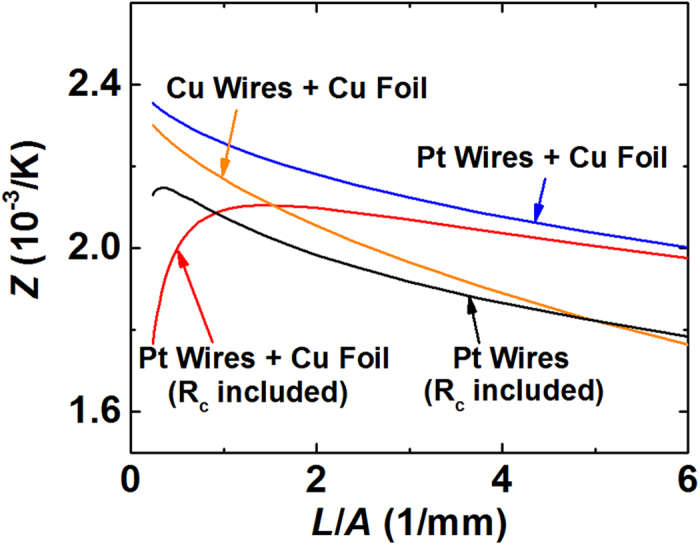
Calculated *Z* of a Bi_2_-Te_3_ based material as a function of the shape factor. The FEM model uses Cu foil either with Cu or Pt wires to simulate the surface current source. To simulate the experimental condition, the FEM model also calculates *Z* including the contact resistance, *R*_*c*_, either with the surface current source or with the point current source.

**Table 1:** Description of Test Materials.

Type	1	2	3
Composition	Bi_2_Te_2.7_Se_0.3_	Bi_0.5_Sb_1.5_Te_3_	
*α* (μV/K)	−201	220	194
*ρ* (mΩcm)	1.30	1.28	1.60
*k* (W/mK)	1.45	1.42	0.98
*Z* (10^−3^/K)	2.14	2.65	2.40
Area, *A* (mm^2^)	7.07		2.9–43.6
Length, *l* (mm)	3.3–35.4		15
Wire Material	Au	Pt	
